# Previous Early Antenatal Service Utilization Improves Timely Booking: Cross-Sectional Study at University of Gondar Hospital, Northwest Ethiopia

**DOI:** 10.1155/2014/132494

**Published:** 2014-07-01

**Authors:** Tadesse Belayneh, Mulat Adefris, Gashaw Andargie

**Affiliations:** ^1^Department of Medical Anesthesiology, College of Medicine and Health Sciences, University of Gondar, Kebele 16, P.O. Box 196, Gondar, Ethiopia; ^2^Department of Gynecology and Obstetrics, College of Medicine and Health Sciences, University of Gondar, P.O. Box 196, Gondar, Ethiopia; ^3^Institute of Public Health, College of Medicine and Health Sciences, University of Gondar, P.O. Box 196, Gondar, Ethiopia

## Abstract

*Background.* Early booking of antenatal care (ANC) is regarded as a cornerstone of maternal and neonatal health care. However, existing evidence from developing countries indicate that lots of pregnant woman begin ANC booking lately. *Objective.* It was aimed to assess timing of ANC booking and associated factors among pregnant women attending ANC clinic at University of Gondar Hospital, 2013. *Methods.* An institution based cross-sectional study design was used to collect data with a face-to-face interview technique. Bivariate and multivariate analysis was used to identify associated factors for early ANC visit using SPSS version 20. *Results.* From total women (N = 369) interviewed, 47.4% were timely booked. Mothers with younger age (AOR = 3.83, 95% CI: 1.89, 10.53), formal education (AOR = 1.06, 95% CI: 1.03, −7.61), previous early ANC visit (AOR = 2.39, 95% CI: 2.23, 9.86), and perceived ANC visit per pregnancy of four and greater were significantly associated with early ANC visit. *Conclusions.* Although late booking is a problem in this study, previous early utilization of ANC visit favors current timely booking. This indicates that the importance of early booking was appropriately addressed from previous visits. Counseling of timely booking during ANC visit should be strengthened. Moreover, empowering through education is also recommended.

## 1. Introduction

World Health Organization (WHO) with a fifth Millennium Development Goal has planned to reduce maternal deaths by three-quarters by the year 2015 [1]. Antenatal care, a care given to pregnant women, is widely used for prevention, early diagnosis, and treatment of general medical- and pregnancy-related complications [2].

Early ANC booking and regular follow-up of services usually provides opportunities for delivering health information and interventions (i.e., via early detection of modifiable preexisting medical conditions like Heart disease, Diabetes Mellitus, Hypertensive disorders, HIV/AIDS, and severe anemia) that can significantly enhance the health of the mother and fetus [3–9]. On the contrary, opportunities to provide information and other interventions pertaining to their reproductive health and the health of their unborn child are missed when a woman initiates ANC in late time of her pregnancy [10, 11].

The new World Health Organization ANC model states that every pregnant woman is at risk of complications and recommends early an ANC visit, of which the first should be during the first trimester. The visit is used to classify pregnant women into two groups based on previous history of pregnancy, current pregnancy state, and general medical conditions. Those eligible to receive routine ANC (basic component) and those who need special care on average account for 25% of all pregnant women initiating ANC [12]. Low ANC coverage, few visits, and late booking are common problems throughout Sub-Saharan Africa posing difficulty in accomplishing the WHO recommendation [13, 14].

According to the Ethiopian Demographic and Health Survey 2011 report, only 11.2% of women had an ANC visit before their fourth month of pregnancy, almost twofold increase from 6 percent in the 2005 EDHS. In urban settings where the health services are physically accessible relative to rural areas, 31.0% of mothers seek the service before four months of gestation and 23.1% did not attend at all. The figure for rural women is much worse than this; that is, 7.7% sought ANC before 16 weeks and 63.1% did not attend ANC at all. In Ethiopia, ANC services are provided free of payment at the governmental health facilities [15]. A similar study done in Addis Ababa showed a 40.0% of first trimester booking with a recommended period [16]. Likewise, study finding in Nairobi, Kenya indicated that 85% of women initiated visits later than the first trimester [17]. These trends have been also noted in other Sub-Saharan African countries [18, 19].

**Figure 1:**
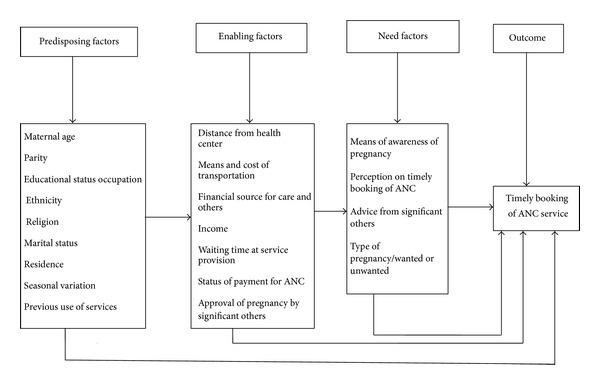
Conceptual framework for timely use of ANC (adapted from Anderson 1995).

Utilization of health services is a complex behavioral phenomenon. Empirical studies of preventive and curative services have often found out that the use of health services is related to the availability, quality, cost of services, social structure, health beliefs, and personal characteristics of the users [20–22]. Reviewed literatures showed that maternal age, marital status, maternal education, occupation, ethnicity, religion, family income, residence, parity, history of abortion, child birth outcome, experience of service utilization, pregnancy-related complications, wanted or unwanted pregnancy, and influence of the husband were predictors that either positively or negatively influence timing of ANC booking (Figure 1) [23–26].

Studies on the prevalence of early ANC visit and associated factors are scanty in Ethiopia and unavailable in the study area. Therefore, this study aimed at giving information about the prevalence of early ANC visit and associated factors in order to help improve the health of mothers and their fetus among pregnant women attending ANC at the University of Gondar Hospital, Northwest Ethiopia.

## 2. Methods

Hospital-based cross-sectional study was conducted from January 1 to February 28, 2013, at the University of Gondar Hospital. The Hospital is a 500-bed capacity teaching hospital founded in Amhara region, Northwest Ethiopia. Medical, gynecologic and obstetrics, pediatrics, and surgical patients are served by the Hospital at outpatient and inpatient levels. It serves for more than 5 million populations in area. According to EDHS 2011, 59.1% of women in Amhara region did not attend ANC at all [15].

The sample size (*N* = 369) was calculated using single population proportion formula based on the following assumptions: taking 40% prevalence on early timing of the first ANC visit at 12 weeks from a study conducted in Addis Ababa [16], 95% confidence interval, and a 5% margin of error.

Study subjects were recruited for the study at ANC service when they appear to begin the service or return for ANC follow-up. Every second case that was willing to participate during the study period was taken until the required sample size was obtained.

Data were collected using a pretested structured questionnaire via trained female nurses who were not from the same facility. Pretesting was conducted outside the study area. Data collectors interviewed pregnant women waiting after they completed their daily visits. The purpose of data collection and the importance of the study as well as the significance of true information were enlightened in order to maximize the response rate and to generate reliable data.

All responses to the questionnaires were coded, entered, and analyzed using SPSS version 20.0.

Variables with *P* value of up to 0.2 in bivariate analysis were entered into the multivariate model. The binary logistic regression method was employed and variables with a *P* value of <0.05 were considered as significantly associated with the early ANC visit. The early ANC visit is considered if pregnant women started their first ANC visit within the first 12 weeks of gestation. *P* value and 95% confidence interval were used to check the statistical significance. The odds ratio was utilized to determine the strength of association between independent variables and ANC booking. Ethical clearance was obtained from the Institutional Review Board of University of Gondar and an official permission letter was secured from the University of Gondar Hospital Clinical Director. Participants were communicated individually about the purpose of the study and verbal informed consent was taken before the interview. The right of participants to withdraw from the study at any time, without any precondition, was kept and disclosed to respondents. The name of participants was not taken for reasons of confidentiality.

## 3. Results 

### 3.1. Sociodemographic Characteristics of the Study Participants

All pregnant mothers (*N* = 369) who attended the service responded to the interview (100%). The mean age of the participants was 24.9 years (±4.1 years). One hundred and forty-two (38.5%) of the participants had secondary school level education and 124 (33.6%) were employed. Almost all 366 (99.2%) participants were married (Table 1).

### 3.2. Timing of Initial ANC Visit

Out of 369 pregnant mothers included in this study, 174 (47.2%) pregnant mothers started their first ANC visit early (CI 1.48–1.58, with mean of 1.52), while the remaining 195 (52.8%) pregnant mothers started ANC late in either second or third trimester. In both cases, the timing of the first ANC booking ranges from 1 month to 8 months of gestation. Similarly, the mean month of initiation of ANC was 4.4 months (SD = 1.4), and the median duration was 5 months.

### 3.3. Obstetric Characteristics of the Participants

As shown in Table 2, among 170 respondents who had a history of pregnancy, 131 (77.1%) of them visited ANC. Of those who visited ANC, 62 (47.3%) of them booked within 12 weeks of pregnancy, while the remaining 69 (52.7%) participants booked after 12 weeks of gestation. The current pregnancy was planned and wanted in the majority (90.0%) of the women.

Regarding to perception of correct time of ANC booking, 256 (69.4%) mother perceived within 12 weeks of gestation, while 113 (30.6%) were after 12 weeks of gestation. Of those who correctly perceived the recommended time, 122 (70.1%) booked early in the current pregnancy.

232 (62.9%) of women perceived that four and more ANC visits were necessary. On the contrary, 137 (37.1%) of them perceived that less than four ANC visits were sufficient throughout the whole pregnancy period (Table 2).

### 3.4. Factors Associated with Early Booking of ANC Visit

The analysis was done using bivariate and multivariate binary logistic regression. The model fitness was checked with Hosmer and Lemeshow test. Results from multivariate analysis indicated that pregnant mothers of younger age, having a higher educational level, previous late booking practice, history of abortion, and the perception of ANC visit per pregnancy, were significantly associated with the early ANC visit. However, the number of children alive, mode of pervious delivery, planned pregnancy, place of ANC visit, pregnancy-related complications in the current pregnancy, history of abortion, having less than five children, and means of approving pregnancy were not significantly associated with the early ANC visit. Accordingly, pregnant mothers at younger age were 3.83 times more likely to book earlier compared to older ones (AOR = 3.89, 95% CI: 1.89, 10.53). Similarly, those having formal education were 1.06 times more likely to book earlier compared to those who cannot read and write (AOR = 1.06, 95% CI: 1.03, −7.61). Those women who booked early in previous pregnancy were 2.39 times more likely to book earlier compared to those who booked lately (AOR = 2.39, 95% CI: 2.23, 9.86). Moreover, those who attended four and greater ANC visits per pregnancy (AOR = 1.39, 95% CI: 1.89, 7.53) were more likely to book earlier compared to their counterparts (Table 3).

## 4. Discussion

Antenatal care is more beneficial in preventing adverse pregnancy outcomes when received early in pregnancy and continued until delivery [27–29]. Early detection of problems in pregnancy leads to timely referrals for women in high-risk categories or with complications; this is particularly true in Ethiopia, where three-quarters of the population lives in rural areas and where physical barriers pose a challenge to providing health care [15].

According to this study, nearly half (47.4%) of the respondents started their ANC within the recommended time and the rest (52.6%) booked late. This result is low compared to the recommendation of WHO which states that each and every pregnant woman should start the first ANC within the first trimester of pregnancy [12]. On the contrary, the finding was high compared to the National EDHS report (11.0%, 2011), ANC coverage in Addis Ababa (40.0%, 2008), Debre Berhan (26.2%, 2012), Hadiya Zone (8.7%, 2010), and (12.5%, 2009) Yem Southern Ethiopia [15, 16, 30–32]. This gap might be because of differences in the study population; that is, this study included urban residents, whereas the others (Debre Berhan, Hadiya, and Yem) in both urban and rural residents. Moreover, this can be influenced by the fact that the study used hospital based setting and therefore they were more likely to attend ANC earlier than the general population. Time gap might also be the other reason. This finding suggests that physical and financial accessibilities of the service alone cannot ensure proper utilization of available service. The result was also higher compared to other African countries, South Western Nigeria (29%, 2008), Tanzania (19%, 2000), Lao People's Democratic Republic (28%, 2010), and (17.4%, 2006) rural Western Kenya [13, 33–35]. However, this result is remarkably lower than the findings from developed and some developing countries, where the vast majority of pregnant women present early for ANC [36, 37].

In the current study, women having formal education were more likely to initiate ANC visit earlier than their counterparts (AOR 1.06, 95% CI: 1.03–7.6) which is similar to a study conducted elsewhere in the developing countries [1, 38, 39]. This could be explained by the fact that women with secondary school or higher education were more likely to be employed have more income than their counterparts. The rationale is that by educating girls especially of secondary school or higher level, they are equipped not only with the right tools to make proper health care decisions, but also with skills that enhance their future financial independence, thereby elevating their status in the communities where they live [38, 39].

The proportion of respondents who have had their first visit within the recommended time in the preceding pregnancy was 47.3%, while 56.9% of these pregnant women who booked ANC within the recommended time in the previous pregnancy were booked within the current pregnancy. Accordingly, those who visited ANC earlier in former pregnancy were more likely to book earlier for their current pregnancy (AOR = 2.39, 95% CI: 2.23, 9.86), showing that past experience of early ANC service utilization demonstrated timely booking in the current visit. This depicted that women were appropriately informed on time of booking from counseling and health education sessions during previous pregnancies. The result of this study is in contradiction with Addis Ababa's finding which showed that previous ANC utilization did not improve timely booking [16].

The result of this study showed that good perception of the initial ANC visit was a factor for an early ANC visit. Women who perceived initiation of ANC visit in the first trimester were more likely to make four or more visits than those who initiated care in the second and third trimesters. This finding was supported by studies done in Addis Ababa [16] and Niger Delta, Nigeria, which showed that the major reason for the late ANC visit was a misconception about the early ANC visit [40].

The current study also showed that age was associated with early booking. Women less than 30 years old were more likely to book for ANC earlier than older women. This finding was similar to those of studies done in Addis Ababa (Ethiopia), Nigeria, Kenya, and India [6, 16, 23, 24]. This might be because young women at their first pregnancy are more careful about their pregnancy and therefore require institutional care more than older women. In addition, younger women tend to be more educated than older ones [2].

It was reported by different researchers that previous obstetric complications such as stillbirth, abortion, eclampsia, intrauterine fetal death, and cesarean section have no influence on gestational age at booking [21, 22]. Similarly, the current study showed that those women who had no history of abortion were more likely to book earlier than women with abortion history.

## 5. Limitations of the Study

As this is a cross-sectional study, the associations observed may not be causal enough, and since this study is institution based it may not be generalisable. Additionally, it only includes the urban clients who have more access to services and information.

## 6. Conclusions and Recommendations

In conclusion, even though the ANC services are available freely to all women, late booking continues to be a problem in the study setting. Maternal education, previous history of early booking, age, and perception on frequency of ANC visit per pregnancy were significantly associated with the early ANC visit. As per the findings of this study, awareness creation and strengthening on the importance of the early ANC visit need to be emphasized at the time of service provision. Empowering mothers through education is also recommended.

## Figures and Tables

**Table 1:** Sociodemographic characteristics of pregnant mothers by time of booking, University of Gondar Hospital, Northwest Ethiopia, 2013 (*N* = 369).

Variables	Frequency (%)
Age	
15–19	24 (6.5%)
20–24	153 (41.5%)
25–29	135 (36.6%)
30–34	46 (12.5%)
35–40	11 (3.0%)
Marital status	
Married	366 (99.2%)
Single	3 (0.8%)
Educational status	
No formal education	72 (19.5%)
Primary (1–8)	74 (20.1%)
Secondary (9–12)	142 (38.5%)
Above secondary	81 (22.0%)
Ethnicity	
Amhara	354 (95.9%)
Tigre	15 (4.1%)
Religion	
Christianity^#^	320 (86.7%)
Muslim	49 (13.3%)
Occupation	
Employed	124 (33.6%)
Nonemployed	245 (66.4%)

Christianity^#^ = orthodox + protestant + catholic.

**Table 2:** Obstetric characteristics of pregnant women by time of booking, University of Gondar Hospital, Northwest Ethiopia, 2013.

Variables	Total (%)
Number of children alive (*N* = 168)	
1-2 children	120 (74.4%)
>2 children	48 (25.6%)
Had ANC visit for last pregnancy (*N* = 170)	
Yes	131 (77.1%)
No	39 (22.9%)
Previous ANC first visit (*N* = 131)	
Booked early (≤4 months)	62 (47.3%)
Booked lately (>4 months)	69 (52.7%)
State of current pregnancy (*N* = 369)	
Planned	332 (90.0%)
Unplanned	37 (10.0%)
History of abortion (*N* = 369)	
Yes	94 (25.5%)
No	275 (74.5%)
History of death of child (*N* = 369)	
Yes	38 (10.3%)
No	331 (89.7%)
Perceptions on timing of first visit (*N* = 369)	
Early (≤12 weeks)	256 (69.4%)
Lately (>12 weeks)	113 (30.6%)
Perceived number of ANC visits per pregnancy (*N* = 369)	
<4 times	137 (37.1%)
≥4 times	232 (62.9%)
Mode of previous delivery (*N* = 166)	
Spontaneous vaginal delivery	143 (86.1%)
C-section	23 (13.9%)

**Table 3:** Factors affecting booking of first ANC visit, University of Gondar Hospital, Northwest Ethiopia, 2013 (*N* = 369).

Variables	Time at first visit		Crude OR (95% CI)	Adjusted OR (95% CI)
	Timely booked *n* (%)	Lately booked *n* (%)		
Educational status				
Cannot read and write	33 (19.0)	39 (20.0)	1	1
Formal education	141 (81.0)	156 (80.0)	3.16∗ (1.03–8.64)	1.06∗ (1.03–7.61)
Age				
16–30	167 (95.9)	168 (86.2)	2.33∗ (1.09–11.91)	3.83∗ (1.89–10.53)
31–45	7 (4.1)	27 (13.8)	1	1
Previous ANC first time visit (*n* = 131)				
Before and at 12 weeks	33 (56.9)	26 (35.6)	2.01∗ (1.12–4.04)	2.39∗ (2.23–9.86)
After 12 weeks	25 (43.1)	47 (64.4)	1	1
History of abortion				
Yes	41 (11.1)	53 (27.2)	1	1
No	133 (36.0)	142 (72.8)	1.01∗ (2.44–5.84)	1.21∗ (2.17–7.94)
Perceived sufficient number of ANC visits/pregnancy				
Less than 4 times	43 (24.7)	61 (31.3)	1	1
More than 4 times	131 (75.3)	134 (68.7)	1.18∗ (1.99–4.26)	1.39∗ (1.89–7.53)
Occupation				
Employed	42 (24.1)	82 (42.1)	1	1
Nonemployed	132 (75.9)	113 (57.9)	3.12^#^ (1.23–12.3)	2.28 (0.71–10.2)^#^
Had ANC visit for last pregnancy (*n* = 170)				
Yes	54 (80.6)	77 (74.8)	0.71 (0.33–1.51)^#^	4.67 (1.34–16.27)^#^
No	13 (19.4)	26 (25.2)	1	1
State of current pregnancy (*N* = 369)				
Planned	154 (85.5)	178 (91.3)	1.36 (0.69–2.69)^#^	4.37 (0.79–24.11)^#^
Unplanned	20 (11.5)	17 (8.7)	1	1

*Significant and ^#^nonsignificant from the multivariate logistic regression.
